# Medium-Chain Fatty Acids from *Eugenia winzerlingii* Leaves Causing Insect Settling Deterrent, Nematicidal, and Phytotoxic Effects

**DOI:** 10.3390/molecules24091724

**Published:** 2019-05-03

**Authors:** Angel Cruz-Estrada, Esaú Ruiz-Sánchez, Jairo Cristóbal-alejo, Azucena González-Coloma, María Fe Andrés, Marcela Gamboa-Angulo

**Affiliations:** 1 Unidad de Biotecnología, Centro de Investigación Científica de Yucatán, Colonia Chuburná de Hidalgo, Mérida C.P. 97200, Yucatán, Mexico; angel_estrad@yahoo.com.mx; 2 Tecnológico Nacional de México, Instituto Tecnológico de Conkal, Conkal C.P. 97345, Yucatán, Mexico; esau.ruiz@itconkal.edu.mx (E.R.-S.); jairoca54@hotmail.com (J.C.-a.); 3 Instituto de Ciencias Agrarias-CSIC, 115 Dpdo-28006 Madrid, Spain; azu@ica.csic.es (A.G.-C.); mafay@ica.csic.es (M.F.A.)

**Keywords:** Bemisia tabaci, decanoic acid, dodecanoic acid, Eugenia winzerlingii, Lolium perenne, Meloidogyne, Myzus persicae, Solanum lycopersicum, undecanoic acid

## Abstract

*Eugenia winzerlingii* (Myrtaceae) is an endemic plant from the Yucatan peninsula. Its organic extracts and fractions from leaves have been tested on two phloem-feeding insects, *Bemisia tabaci* and *Myzus persicae*, on two plant parasitic nematodes, *Meloidogyne incognita* and *Meloidogyne javanica*, and phytotoxicity on *Lolium perenne* and *Solanum lycopersicum*. Results showed that both the hexane extract and the ethyl acetate extract, as well as the fractions, have strong antifeedant and nematicidal effects. Gas chromatography-mass spectrometry analyses of methylated active fractions revealed the presence of a mixture of fatty acids. Authentic standards of detected fatty acids and methyl and ethyl derivatives were tested on target organisms. The most active compounds were decanoic, undecanoic, and dodecanoic acids. Methyl and ethyl ester derivatives had lower effects in comparison with free fatty acids. Dose-response experiments showed that undecanoic acid was the most potent compound with EC_50_ values of 21 and 6 nmol/cm^2^ for *M. persicae* and *B. tabaci*, respectively, and 192 and 64 nmol for *M. incognita* and *M. javanica*, respectively. In a phytotoxicity assay, medium-chain fatty acids caused a decrease of 38–52% in root length and 50–60% in leaf length of *L. perenne*, but no effects were observed on *S.* *lycopersicum*. This study highlights the importance of the genus *Eugenia* as a source of bioactive metabolites for plant pest management.

## 1. Introduction

Plant pests are a severe constraint in crop production worldwide. Various species of insects and nematodes have primary importance due to the enormous losses they cause in crop productivity [1,2]. The sap-feeding and virus-transmitting whitefly, *Bemisia tabaci* (Hemiptera: Aleyrodidae), and aphid, *Myzus persicae* (Hemiptera: Aphididae), are two of the most damaging phytophagous insects in vegetable crops [3,4]. Likewise, the plant parasitic nematodes *Meloidogyne incognita* and *Meloydogine javanica* are among the most important species of root-knot nematodes in horticultural and field crops, while the population establishment of *B. tabaci* and *M. persicae* directly affects leaf-related physiological processes and the colonization of *Meloidogyne* spp. impairs the function of plant roots, reducing nutrient and water uptake, resulting in poor plant growth and a decline in fruit yield [5].

Crop protection against parasitic nematodes and phytophagous insects has been historically addressed through the use of synthetic pesticides. However, pesticide resistance and environmental pollution have led to the search for biorational strategies to manage plant pests [6,7,8]. Plant-derived products are considered to be environmentally friendly and represent cost-effective alternatives to control phytophagous insects and plant pathogenic microorganisms [9,10,11]. Moreover, an extensive number of studies have reported the potential of a wide range of plant-derived metabolites against insects and nematodes [12,13,14]. The screening of new biologically active compounds from new sources is a key step in the development of more active compounds [15].

**Figure 1:**
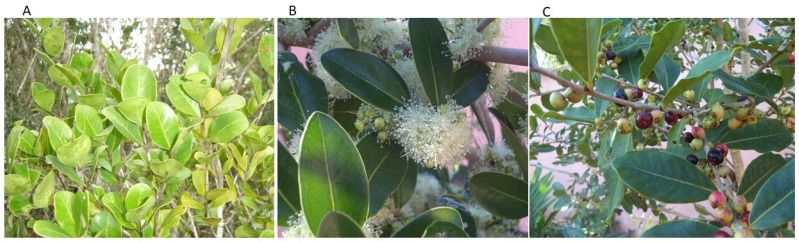
(**A**) *Eugenia winzerlingii* Standl., shrub in field, (**B**) flowers, and (**C**) fruits.

The genus *Eugenia* is one of the largest members of Myrtaceae family distributed in tropical and subtropical regions from Mexico to Central and South America, with estimations of 1000 species [16,17]. In Mexico, 81 species have been documented from the genus *Eugenia* [18]. Previous studies by our group have found that the organic extracts from *Eugenia winzerlingii* Standl. leaves have strong oviposition deterrent effects on the whitefly *Bemisia tabaci* (Homoptera: Aleyrodidae) and have a lethal effect on the root-knot nematode *Meloidogyne incognita* [19,20]. The species *E. winzerlingii* (Figure 1) is a shrub that grows in the Yucatan peninsula, Belize, and Guatemala [21]. To our knowledge, no purification or identification of the active constituents of *E. winzerlingii* have been carried out thus far.

Secondary metabolites of leaves, barks, seeds, and fruits from approximately 350 *Eugenia* species, have diverse biological activity, including analgesic, antioxidant, antifungal, antimicrobial, antidiabetic, anti-inflammatory, and cytotoxic [16,22]. Various studies have reported that essential oils of the leaves of *Eugenia* spp. and their constituents are active against bacteria, fungi, and protozoan [16,22,23,24,25,26]. However, studies focusing on the effects of extracts or metabolites on plant pests are scarce [27,28]. The toxicity of essential oils and organic extracts of *Eugenia caryophyllata* leaves has been reported in the human ectoparasite *Pediculus capitis* [29], the mosquito *Aedes aegypti* [30], the stored grain pests *Acanthoscelides obtectus*, *Sitophilus zeamais*, and *Tribolium castaneum* [31,32] and the plant feeders *Oryctes agamemnon*, *Pochazia shantungensis*, and *Trialeurodes vaporariorum* [33,34,35], and in the nematodes *Bursaphelenchus xylophilus* and *Meloidogyne incognita* [36]. In addition, essential oils from *Eugenia uniflora* have shown strong repellent effects on *Diaphania hyalinata* [37].

In this paper, in the search for novel plant-derived metabolites from regional plants, we have identified the active components causing insect deterrent and nematicidal activity of the organic extracts from *E. winzerlingii* leaves using a partial bioassay-guided fractionation of the hexane and ethyl acetate extracts. In the bioassays, four economically important plant pest species were used: The sap-feeding insects *B. tabaci* and *M. persicae* and the root-knot nematodes *M. incognita* and *M. javanica.* In addition, the phytotoxicity of the identified compounds was assessed on *Lolium perenne* L. (Apoaceae) and *Solanum lycopersicum* (Solanaceae).

## 2. Results

### 2.1. Effect of Extracts and their Fractions on Insect Settling Deterrence and Nematode Mortality

The hexane (HEX) and ethyl acetate (ETA) extracts obtained from *E. winzerlingii* leaves showed a strong settling inhibition (SI) of *M. persicae* (SI, 87.9–96.9%) and mortal (M) effect on second stage juveniles (J2) of *M. incognita* and *M. javanica* (M, 100%). Chromatographic separation of the HEX and ETA extracts yielded six (1a–1f) and four (2a–2d) fractions, respectively. Among the fractions obtained, 1b, 1c, 1d, 2b, and 2c showed the strongest SI effect on *M. persicae* (>90%); while 1b, 1c, 2b, and 2c fractions also had the strongest effect on *M. incognita* and *M. javanica* (M, 100%) (Table 1).

### 2.2. Major Constituents of Active Fractions

The most active fractions 1b and 1c obtained from the HEX extract and 2b and 2c fractions from ETA extract were analyzed by gas chromatography–mass spectrometry (GC-MS). All fractions contained a mixture of saturated fatty acids identified as decanoic, undecanoic, dodecanoic, tridecanoic, and tetradecanoic acids. In order to verify the presence of these and other fatty acids in the fractions, their methyl esterifed derivatives were obtained. The most abundant component was dodecanoic acid (32.41–43.56%) in all active fractions. The fraction 1c did not contain the first two medium-chain fatty acids. The long-chain fatty acid, hexadecanoic acid, was present in all fractions except 2c (Table 2; Figures S1 and S2).

### 2.3. Biological Activity of Fatty Acids and Derivatives

All saturated fatty acids and their methyl/ethyl ester derivatives were evaluated on insect SI, oviposition inhibition (OI), and nematode mortality. Overall, methyl ester derivatives had similar or slightly lower effects in comparison with free fatty acids, whereas ethyl derivatives had significantly lower effects, particularly on *M. javanica* (Table 3). Decanoic, undecanoic, and dodecanoic acids had the greatest effect on insect SI. Values for SI of *M. persicae* were 83.3–97.6% and for OI of *B. tabaci* they were 94.3–98.2%. All fatty acids also showed strong lethal effects on *M. incognita* and *M. javanica* J2 (M = 94.4–100%) (Table 3).

**Figure 2:**
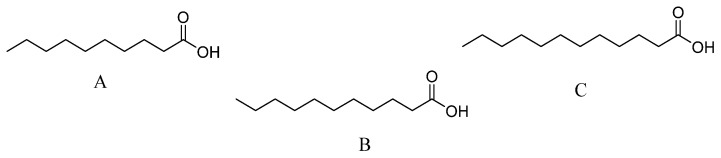
The most active medium-chain fatty acids from *Eugenia winzerlingii* leaves, (**A**) decanoic acid, (**B**) undecanoic acid, and (**C**) dodecanoic acid.

Dose-response experiments to calculate the median effective concentrations (EC_50_) indicated that undecanoic acid was the most potent compound on all target organisms (Table 4, Figure 2).

### 2.4. Phytotoxicity of Fatty Acids

The evaluation of phytotoxicity of the most active medium-chain fatty acids on two plant species showed that none of the compounds affected germination or rootlet growth of the dicotyledonous *Solanum lycopersicum*. Similarly, HEX extract had no effect on these variables. In contrast, ETA extract caused a slight effect on the rootlet length of *S. lycopersicum*. All fatty acids caused a significant decrease in growth of rootlet and leaves of the monocotyledonous *Lolium perenne*. In addition, germination was also affected by undecanoic acid (Table 5).

## 3. Discussion

In the search for novel plant-derived compounds for pest management, we have found that the organic extracts from *E. winzerlingii* leaves were promissory as insect antifeedant and nematicidal agents [19,20]. In the present work, we have shown that the ETA and HEX extracts from *E. winzerlingii* leaves, as well as fractions 1b, 1c, 2b, and 2c showed strong anti-settling effects on the aphid *M. persicae* and nematicidal effects on *M. incognita* and *M. javanica* (Table 1). In a previous work, we documented that these extracts also caused a high mortality rate in nymphs (LC_50_ of 0.25–0.78 mg/mL) of *B. tabaci*, and ETA extract had good oviposition inhibition effects on adults (EC_50_ of 27.86 µg/cm^2^) [19]. Furthermore, among 20 native plant species tested, the total ethanol extract of *E. winzerlingii* leaves was the most effective on J2 *M. incognita* with a median effective doses (ED_50_) of 133.4 µg/mL [20]. In comparison with studies using essential oils of other species of *Eugenia*, the organic extracts from *E. winzerlingii* leaves have a similar potency to that of the essential oils from *E. uniflora* leaves (10 µg/µL), as observed in oviposition deterrence and larval repellence of the Lepidopteran *Dyapahania hyalinata* [37]. In contrast, a higher potency of essential oils of *E. caryophyllata* leaves (0.4 µg/µL) was observed in the Hemipteran *Pochazia shantungensis* [34].

The most active fractions 1b, 2b, and 2c contained a mixture of saturated medium-chain (decanoic, undecanoic, and dodecanoic acids) and long-chain (tridecanoic, tetradecanoic, and hexadecanoic acids) fatty acids (Table 2). This is the first report of the effect of a *Eugenia* species on the aphid *M. persicae* and the parasitic nematode *M. javanica*. To our knowledge, isolation and biological activity of medium-chain fatty acids from the *Eugenia* genus have not been previously reported. Thus far, phytochemical studies of other *Eugenia* species with pesticidal properties have reported the presence of caryophyllene oxide, cineol, cymene, elemene, eudesmol, eugenol, furanodiene, furanoelemene, germacrene, linalool, phytol, Selina-1,3,7(11)-trien-8-one, and terpinene, as the most abundant metabolites in essential oils of leaves [22,23,24,25,26,27,37,39,40,41]. It was surprising to find that *E. winzerlingii* leaves do not biosynthesize essential oils.

Plant-derived medium-chain fatty acids are mainly found in the seeds of oleaginous species [42]. These kinds of metabolites are also ubiquitous in plant cuticle waxes, where they are more likely to play an important role at the interface between plants and phytophagous insects [43]. Various studies provide evidence that the exogenous application of fatty acids alters the settling or feeding behavior of insects. Fatty acids can either deter or attract insects, depending on their structures and lengths. For example, saturated medium-chain (decanoic, undecanoic, and dodecanoic acids), and long-chain unsaturated fatty acids (oleic, linoleic, and linolenic acids) strongly inhibited settling by the aphid *M. persicae*, but long-chain saturated fatty acids (palmitic and stearic acids) showed the opposite effect [38,44].

In the present study, decanoic, undecanoic, and dodecanoic acids caused oviposition inhibition in *B. tabaci*, which had not previously been reported. Undecanoic acid inhibited settling in *M. persicae* with the same potency that decanoic and dodecanoic acids (25 and 21 nmol, respectively), as previously reported [38,45]. In agreement with our results, deterrent effects of medium-chain fatty acids have been observed in other insect species, such as the mosquitoes *Aedes aegypti*, *Anopheles albimanus*, *Anopheles quadrimaculatus*, and *Culex quinquefasciatus* [46,47,48] and the aphid *Rhopalosiphum maidis* and the beetle *Leptinotarsa decemlineata* [38]. The electrophysiological response of insects to fatty acids causing anti-feeding or oviposition deterrence effects has been related to the stimulation of species-specific sensilla in the antennae [48,49]. However, the mechanisms of how these compounds interact with odor receptors and gustatory receptors have not been studied.

In this work, the fractions that caused insect settling deterrence also showed lethal effects on *M. incognita* and *M. javanica*. Consistent with our results, studies by other authors have demonstrated the nematicidal activity of fatty acids [11,50,51]. In comparison to those studies, we have reported lower EC_50_ values for decanoic and dodecanoic acids on *M. incognita* juveniles [51,52]. The actions of fatty acids on nematodes have been associated with the disruption of cell membranes, which leads to alterations in membrane permeability and ion transport, which in turn compromises internal ion and water homeostasis [50]. In addition, fatty acids may also alter chemotactic behavior in nematodes, which results in a substantial decrease in nematode infection [52].

It is worth mentioning that potassium or sodium salts of fatty acids have shown high efficacy not only on insect deterrence, but also on insect mortality [53,54]. Salts of fatty acids, either alone or in combination with free fatty acids, may kill insects by suffocation and through changes of cuticle and cell permeability [54].

Even though fatty acids have shown the potential to manage insects and nematodes in agriculture, it is important to determine whether the use of these compounds is safe for plants and, therefore, feasible for use as plant protection agents. In this regard, we observed that decanoic, undecanoic, and dodecanoic acids may be phytotoxic to some plant species, given that they reduced germination and root length of *L. perenne*, but caused no effect on *S. lycopersicum*. Toxic effects of fatty acids on plant germination have been documented in various studies [55,56]. The effects of medium-chain fatty acids on seed germination have been linked to changes in the physical properties of membranes, which alters the kinetics of their associated enzymes, affecting water potential and ionic exchange of the embryonic axes [57,58]. We suggest that prior to the use of any fatty acid as a plant protection agent, phytotoxicity tests must be carried out on the plant species in question.

In summary, extracts from *E. winzerlingii* leaves had strong deterrent effects on two phytophagous insects *B. tabaci* and *M. persicae*, as well as nematicidal effects on *M. incognita* and *M. javanica*. The active fractions of the extracts contained a mixture of saturated fatty acids. Evaluation of authentic standards of the identified compounds showed that decanoic, undecanoic, and dodecanoic acids were the most active against all species evaluated. This study contributes to the search for biorational agents for sustainable plant pest management and enriched the phytochemistry knowledge of *E. winzerlingii.* Further investigation is needed in order to evaluate the potential for phytotoxicity of these compounds in relevant plant species.

## 4. Materials and Methods

### 4.1. Plant Material and Extraction

Fresh leaves of *E. winzerlingii* were collected in Calakmul, Campeche, México (coordinates 12° 36′ 15.1″ SE 49° 16′ 0.7″ W). A voucher specimen (PS3021) was deposited at the herbarium “Roger Orellana Lanza” of the Unidad de Recursos Naturales of Centro de Investigación Científica de Yucatán. Leaves were dried under artificial light (50–60 °C) for three days and were ground [19].

Ground leaves (4 kg) were extracted with hexane (3 × 4 L, 24 h, at room temperature). After this process, the residual plant material was subjected to extraction with ethyl acetate, as carried out for hexane extraction. The solvent from the extractions was eliminated by a low-pressure vacuum at 40 °C in a rotary evaporator (Ika RV 10 Control; Ika Works, Inc., Staufen, Germany). The yield of each extract relative to the initial dry mass of ground leaves used was 0.4% for the hexane extract and 2.1% for the ethyl acetate extract.

### 4.2. Chromatographic Fractionation of Organic Extracts 

The hexane extract (14.0 g) was fractionated through a vacuum liquid column (3 cm diameter) packed with silica gel 60 (Merck, Darmstadt, Germany) and eluted with mixtures of hexane-acetone (250 mL, each) of increasing polarity, to yield six fractions (1a–1f). The ETA extract (37.7 g) was filtered with ethyl acetate by clay Tonsil® (Bleaching Earths, Clariant, Moosburg, Germany). The concentrated filtrate (8.5 g) was fractionated through silica gel packed in a vacuum liquid column (3 cm diameter) and eluted with mixtures of hexane-ethyl acetate (200 mL each) mixture of increasing polarity to yield four fractions (2a–2d) [59]. Pre-coated silica gel plates (Kieselgel 60 F_254_, 0.25 mm; Merck) were used for thin layer chromatography (TLC). Detection in the TLC was achieved under ultraviolet light by spraying it with a phosphomolybdic acid reagent, followed by heating for 5 min at 105 °C.

### 4.3. Preparation of Methyl and Ethyl Esters 

Prior to Gas Chromatography-Mass Spectrometry (GC-MS) analysis, active fractions and commercial decanoic acid, undecanoic acid, dodecanoic acid, tridecanoic acid, and tetradecanoic acid (purity > 98%; Sigma–Aldrich, St. Louis, MI, USA) were methylated and ethylated. Samples (30 mg) were diluted with 10 mL acidified methanol or ethanol (1% sulfuric acid) and stirred for 12 h at room temperature. The product of the reaction was suspended in an aqueous solution of 20% Na_2_CO_3_ (20 mL) and extracted with CHCl_3_ (3×, 20 mL, each one) and the solvent was eliminated under vacuum until dry. These derivatives were stored at 4 °C until use in assays [60].

### 4.4. Analytical Method

Analysis of the esterified fractions and esterified commercial standards were performed by GC-MS in an Agilent 6890N Network Gas Chromatograph coupled to an Agilent 5975B Mass selective detector (Santa Clara, CA, USA). Samples (0.5 µL, 1% dichloromethane solution) were injected in an Ultra 1 Agilent GC capillary column (dimethylpolysiloxane, 25 m × 0.32 mm, 0.52 µm film thickness). Helium was used as a carrier gas at a flow rate of 1 mL/min, and the column temperature was initially held at 150 °C for 10 °C/min, then raised to 280 °C for 20 min. Major individual components were identified by comparing their mass spectra with those of the spectrometer database using the NIST 05 library and by comparison with commercial standards.

### 4.5. Bioassays

All fractions obtained from HEX and ETA extracts were evaluated on the settling inhibition of *M. persicae* and mortality of J2 *M. incognita* and *M. javanica*. The activity of authentic standards of the medium-chain fatty acids present in the active fractions and their methylated and ethylated esterified derivatives, obtained as described above, were evaluated in the same three targets as well as on *B. tabaci*. 

#### 4.5.1. Evaluation of settling inhibition on *Myzus persicae*

A colony of *M. persicae* was reared on bell pepper (*Capsicum annuum*) and maintained in a growth chamber at 24 ± 2 °C, 60–70% relative humidity and a photoperiod of 16L:8D. The settling bioassay was conducted in a choice experiment, as described by Castillo [44]. Leaf disks (2 cm^2^) were cut from a fully expanded *C. annuum* leaf, divided into two half-disks, and placed on the bottom of a plastic box (3 cm × 3 cm × 1.5 cm) with a ventilated lid and a bottom lining of 2% agar. An aliquot (10 µL) of extracts and fractions (10 µg/µL), or authentic fatty acid standards (5 µg/µL) dissolved in acetone:water (8:2), were deposited on the surface of one of the half-disks. For control, a second half-disk received only the solvent mixture. After solvent evaporation, 10 newly emerged adults of *M. persicae* were transferred to each box. Twenty replicates were used for each treatment. The boxes were incubated at 22 °C and a photoperiod of 18L:6D. The number of aphids settled on the treated and control leaf sections were recorded after 24 h. The percentage of settling inhibition (SI) was calculated by the formula:

SI = [1 − (%*T*/%*C*)] × 100 (1)

where *T* and *C* are the number of insects settled on the treated and control leaf sections, respectively [57]. For serial dilutions, extracts or fractions were evaluated at 100, 50, and 25 µg/cm^2^, while authentic standards were evaluated at 50, 25, and 12.5 µg/cm^2^.

#### 4.5.2. Evaluation of oviposition inhibition on *Bemisia tabaci*

Deterrence effects on *B. tabaci* oviposition was only evaluated with authentic standards of medium-chain fatty acids. The effects of extracts have been reported elsewhere [19].

A whitefly colony was reared on habanero pepper (*Capsicum chinense*) within entomological cages (1.44 m^3^) in a greenhouse at 25–35 °C, 55–75% relative humidity, and a photoperiod of 12L:12D. The colony was maintained for several years and has been used in previous studies [61]. Oviposition deterrence was evaluated as described by Baldin et al. [62], with some modifications. Leaf sections (2 cm^2^) were cut and treated at the same concentrations as described for *M. persicae*. Single treated and control leaf sections were set on the bottom of a Petri dish (3 cm diameter). After solvent evaporation, 16 unsexed adults (3–5 days after emergence) were transferred to each Petri dish and incubated at 26 ± 2 °C, 75 ± 8% relative humidity and a photoperiod of 16L:8D. Five replicates (Petri dishes) were used for each treatment. The number of eggs laid on each leaf section was recorded 24 h after adult transfer. The percentage of oviposition inhibition (OI) was calculated using the equation:

OI = [1 − (*T*/*C*)] × 100 (2)

where *T* and *C* are the number of eggs on the treated and control leaf sections, respectively. 

#### 4.5.3. Nematicidal Assay

The colonies of *M. incognita* and *M. javanica* were maintained on tomato plants (var. Marmande) and cultivated in plastic pots in a growth chamber at 25 ± 1 °C and 70% relative humidity. For bioassays, egg masses from infected tomato roots were incubated in distilled water at 25 °C for 24 h to obtain second-stage juveniles (J2). Nematicidal activity was evaluated in 96-microwell plates as previously described by Andrés [36] with some modifications. Experimental microwells received a sample (5 µL) of extracts or fractions (20 µg/µL), or fatty acids (10 µg/µL) dissolved in DMSO:0.5%-Tween 20. Subsequently, 95 µl of distilled water containing 100–150 juveniles were added in each well, with a final concentration of 1 µg/µL and 0.5 µg/µL, respectively. Control wells received a 5 µL of solvent mixture. All treatments were replicated four times. Plates were covered to prevent evaporation and kept in darkness at 25 °C, for 72 h. Dose-response experiments were conducted using concentrations that ranged from 1 to 0.0312 µg/µL.

Mortality of juveniles was recorded with the aid of a stereoscopic microscope at 40× after 72 h. The percentages of juvenile mortality in the microwell assays were corrected by elimination of the natural death/mortality of the control:

M = [(% mortality in experimental microwells − % mortality in control microwells)/(100 − % mortality in control microwells) × 100].(3)

#### 4.5.4. Phytotoxic Assay

Phytotoxicity of HEX, ETA, and active fatty acids were tested on *L. perenne* L. and *S. lycorpersicum* [63]. Samples (20 µL) of fatty acids (5 µg/µL) and organic extracts (10 µg/µL) solutions were added to 2.5 diameter filter paper (Whatman grade 2) and placed on 12 well plates. After solvent evaporation, groups of 10 seeds and 500 µL H_2_O were added to each well. Control filter paper received only the solvent. Juglone (5 µg/µL) was included as a positive control. Plates were incubated in a growth chamber (24 ± 2 °C, 16:8 L:D). Seed germination was measured daily for seven days for *L. perenne* and five days for *S. lycopersicum*. Germination was considered successful when radical emerge by rupturing the seed coat. The length of rootlet and leaf were measured by the digitalization of pictures (ImageJ v1.43; http://rsb.info.nih.gov./ij/) at the end of the experiment in 20–26 plantlets randomly selected for each treatment. 

### 4.6. Data Analysis

Data on percentage M, OI, SI were analyzed by one-way ANOVA. Prior to running the data, these were arc-sin transformed in order to satisfy the ANOVA assumption of homogeneity of variance. Mean separation was performed by the Tukey post hoc test, α 0.05. The EC_50_ values were calculated by the Probit analysis. Phytotoxic effects were analyzed by the Mann–Whitney test. All analyses were conducted in the Statistical Analysis System (SAS) software (SAS Institute, Cary, NC, USA), version 8.1 for Windows. Mean differences were considered significant if *p* < 0.05.

## 5. Patents

A part of this work resulted in a patent, which is being evaluated by the Instituto Mexicano de protection intellectual.

## Figures and Tables

**Table 1:** Effect of extracts and fractions from *Eugenia winzerlingii* leaves on *Myzus persicae* (100 µg/cm^2^) and on two root-knot nematodes *Meloidogyne incognita* and *Meloidogyne javanica* (1 µg/µL).

Extracts/Fractions	% SI	% M	
	*M. persicae*	*M. incognita*	*M. javanica*
Hexane extract	87.9 ± 2.1 ab	100 ± 0.0 a	100 ± 0.0 a
1a	60.3 ± 5.6 c	25.0 ± 9.2 c	5.53 ± 0.92 d
1b	97.6 ± 1.4 a	100 ± 0 a	100 ± 0.0 a
1c	94.4 ± 2.1 a	100 ± 0 a	100. ± 0.0 a
1d	90.9 ± 2.2 ab	32.9 ± 7.8 b	3.92 ± 1.13 d
1e	60.4 ± 7.7 c	0 ± 0 d	4.13 ± 0.52 d
1f	70.4 ± 6.9 bc	0 ± 0 d	3.92 ± 0.46 d
Ethyl acetate extract	96.9 ± 1.2 a	100 ± 0 a	100 ± 0.0 a
2a	nt	20.7 ± 13.6 b	11.39 ± 3.3 b
2b	97.6 ± 1.1 a	100 ± 0 a	100.0 ± 0.0 a
2c	91.3 ± 2.7 b	100 ± 0 a	100.0 ± 0.0 a
2d	89.7 ± 2.1 b	0 ± 0 c	13.47 ± 4.8 b

Mean of settling inhibition ± standard error (% SI ± SE) and mortality (% M ± SE) followed by the same letter within the same column are not significantly different (Tukey, *p* < 0.05); nt = not tested.

**Table 2:** Chemical composition found in the methylated fractions of organic extracts from *Eugenia winzerlingii* leaves by gas chromatography–mass spectrometry (GC-MS) analysis.

Compound	Retention Time (min)	Fractions (%)			
		1b	1c	2b	2c
Methyl decanoate	7.27	4.75		6.55	7.08
Methyl undecanoate	8.59	17.24		17.46	17.89
Methyl dodecanoate	9.99	40.22	32.41	43.56	37.81
Methyl tridecanoate	11.25	10.42	12.29	13.67	10.58
Methyl tetradecanoate	12.41	9.07	12.51	12.77	7.51
Methyl hexadecanoate	14.52	2.18	4.08	2.46	
4,8,12,16-Tetramethylheptadecan-4-olide	18.24		8.7		
Unknown	22.04		17.02		
Unknown	22.21	16.1	5.90		
Unknown	27.61				7.50

Compounds were identified by comparison to the NIST05 chemical database and commercial standards. Only compounds with a probability of matching mass spectra > 0.9 are shown.

**Table 3:** Effect of fatty acids and ester derivatives on deterrence of two sap-sucking insects, *Myzus persicae* and *Bemisia tabaci* (50 µg/cm^2^), and mortality of two root-knot nematodes, *Meloidogyne incognita* and *Meloidogyne javanica* (0.5 µg/µL).

Compound	% SI	% OI	% M	
	*M. persicae*	*B. tabaci*	*M. incognita*	*M. javanica*
Decanoic acid	89.7 ± 2.5 ab	98.2 ± 1.7 a	100 ± 0 a	100.0 ± 0.0 a
Undecanoic acid	83.3 ± 3.4 ab	94.3 ± 3.3 a	100 ± 0 a	100.0 ± 0.0 a
Dodecanoic acid	97.6 ± 1.4 a	94.7 ± 2.9 a	100 ± 0 a	94.40 ± 0.4 b
Tridecanoic acid	53.8 ± 8.9 c	Nt	94.4 ± 6.4 a	nt
Tetradecanoic acid	76.2 ± 4.7 b	59.1 ± 9.2 b	100 ± 0 a	90.86 ± 2.5 b
Methyl decanoate	36.3 ± 8.8 fg	10.1 ± 6.2 de	100 ± 0 a	98.7 ± 0.5 a
Methyl undecanoate	33.8 ± 7.1 fg	15.7 ± 14.2 cde	nt	95.4 ± 1.0 b
Methyl dodecanoate	48.8 ± 7.1 efg	0.0 ± 0 e	100 ± 0 a	14.53 ± 0.61 c
Methy tridecanoate	57.7 ± 8.2 cdef	Nt	74.8 ± 5.8 b	nt
Methyl tetradecanoate	93.3 ± 0.2 ab	16.4 ± 10.2 bcde	100 ± 0 a	10.24 ± 1.9 cd
Ethyl decanoate	34.4 ± 6.9 fg	30.7 ± 16.2 bcde	55.9 ± 11.7 b	5.91 ± 0.3 de
Ethyl undecanoate	37.4 ± 34.1 fg	46.6 ± 11.9 bcd	53.9 ± 2.1 b	16.71 ± 3.2 c
Ethy dodecanoate	52.7 ± 7.6 defg	62.9 ± 12 ab	61.6 ± 3.9 b	3.28 ± 0.2 e
Ethyl tetradecanoate	77.4 ± 4.3 b	42.2 ± 13.6 bcd	63.8 ± 3.6 b	3.64 ± 0.4 e

Mean of settling inhibition (% SI ± SE), oviposition inhibition (% OI ± SE), and mortality (% M ± SE) followed by the same letter within the same column are not significantly different. One-way ANOVA followed by Tukey´s multiple comparisons test (*p* < 0.05); nt = not tested.

**Table 4:** Median effective concentrations (EC_50_) of fatty acids on settling deterrence of two sap-sucking insect species, *Myzus persicae* and *Bemisia tabaci*, and median lethal concentration (LC_50_) of two root-knot nematodes, *Meloidogyne incognita* and *Meloidogyne javanica*.

Compound	EC_50_ (nmol/cm^2^)		LC_50_ (nmol/mL)	
	*M. persicae*	*B. tabaci*	*M. incognita*	*M. javanica*
Decanoic acid	*	95 (91–99) a	229 (218–240) a	85 (83–87) b
Undecanoic acid	21 (11–30) a	6 (2–11) c	192 (186–199) b	64 (62–66) c
Dodecanoic acid	*	49 (42–55) b	231 (220–241) a	368 (323–410) a

* Data reported by Santana et al. [38]. EC_50_ and LC_50_ values followed by the same letter within the same column are not significantly different.

**Table 5:** Phytotoxic effects of fatty acids (50 µg/cm^2^) and extracts (100 µg/cm^2^) on *Lolium perenne* and *Solanum lycopersicum*.

Extract	*Lolium perenne ^a^*			*Solanum lycopersicum ^a^*	
Fatty Acid	Germination	Root Length	Leaf Length	Germination	Rootlet Length
Hexane extract	62.16 ± 9.62	61.21 ± 8.05	81.36 ± 10.30	102.56 ± 0.0	94.49 ± 8.64
Ethyl acetate extract	72.97 ± 11.47	76.79 ± 8.61	80.83 ± 8.22	97.44 ± 7.52	67.96 ± 5.46
Decanoic acid	72.2 ± 10.1	62.3 ± 11.0 *	50.5 ± 10.4 *	125.8 ± 4.6	88.1 ± 12.0
Undecanoic acid	58.3 ± 7.5 *	48.1 ± 11.0 *	39.2 ± 13.4 *	129.0 ± 0.0	87.1 ± 11.7
Dodecanoic acid	94.4 ± 4.5	58.7 ± 8.7 *	46.4 ± 9.2 *	122.6 ± 9.3	118.3 ± 13.2

^a^ Percentage of control. Values are means (± SE). * indicate significant differences of their own control (*p* < 0.05, Mann Whitney test).

## Supplementary Materials

The following are available online at https://www.mdpi.com/1420-3049/24/9/1724/s1, Figure S1: Gas chromatogram of fractions obtained from hexane extract of *Eugenia winzelingii* leaves, Figure S2: Gas chromatogram of fractions obtained from ethyl acetate extract of *Eugenia winzelingii* leaves.
