# Supplementary figures and images for: Medium-Chain Fatty Acids from Eugenia winzerlingii Leaves Causing Insect Settling Deterrent, Nematicidal, and Phytotoxic Effects
Source: Molecules. 2019 May 3;24(9):1724. doi: 10.3390/molecules24091724 (PMC6540168; doi:10.3390/molecules24091724)

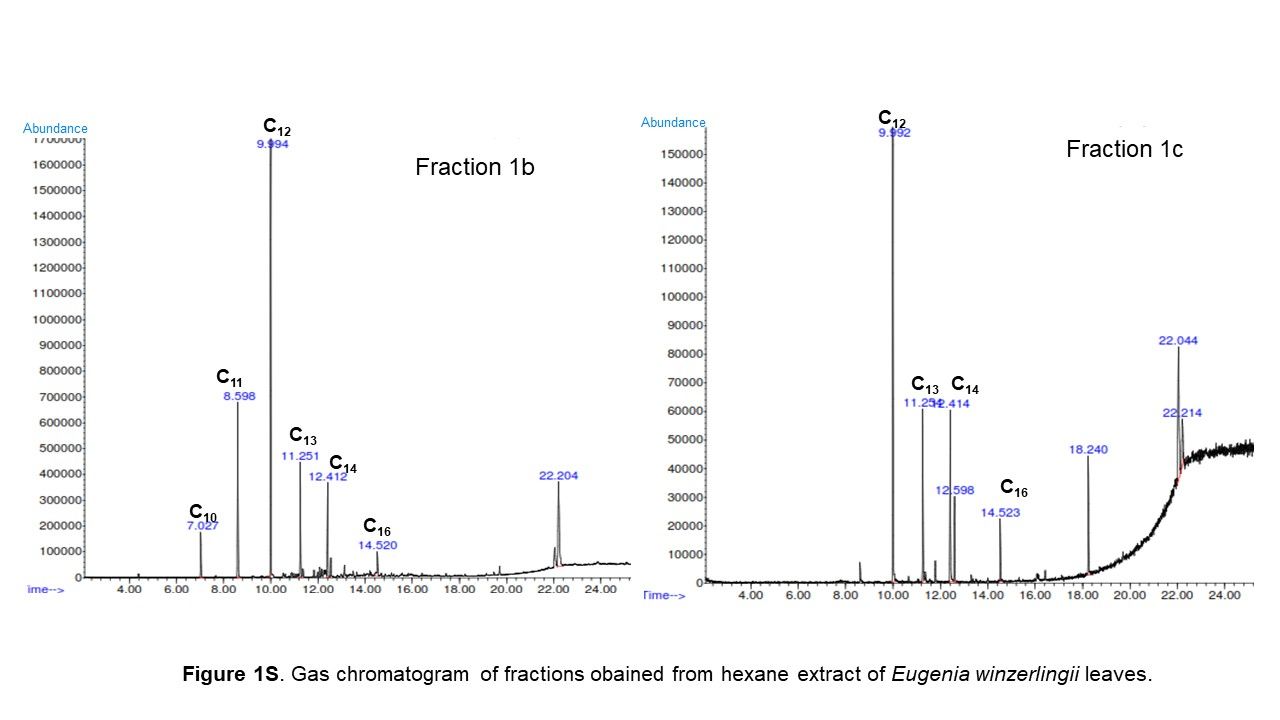

Supplement: Supplementary file 1 [file molecules-24-01724-s001.zip › Suplementary material/Figure 1S.jpg]

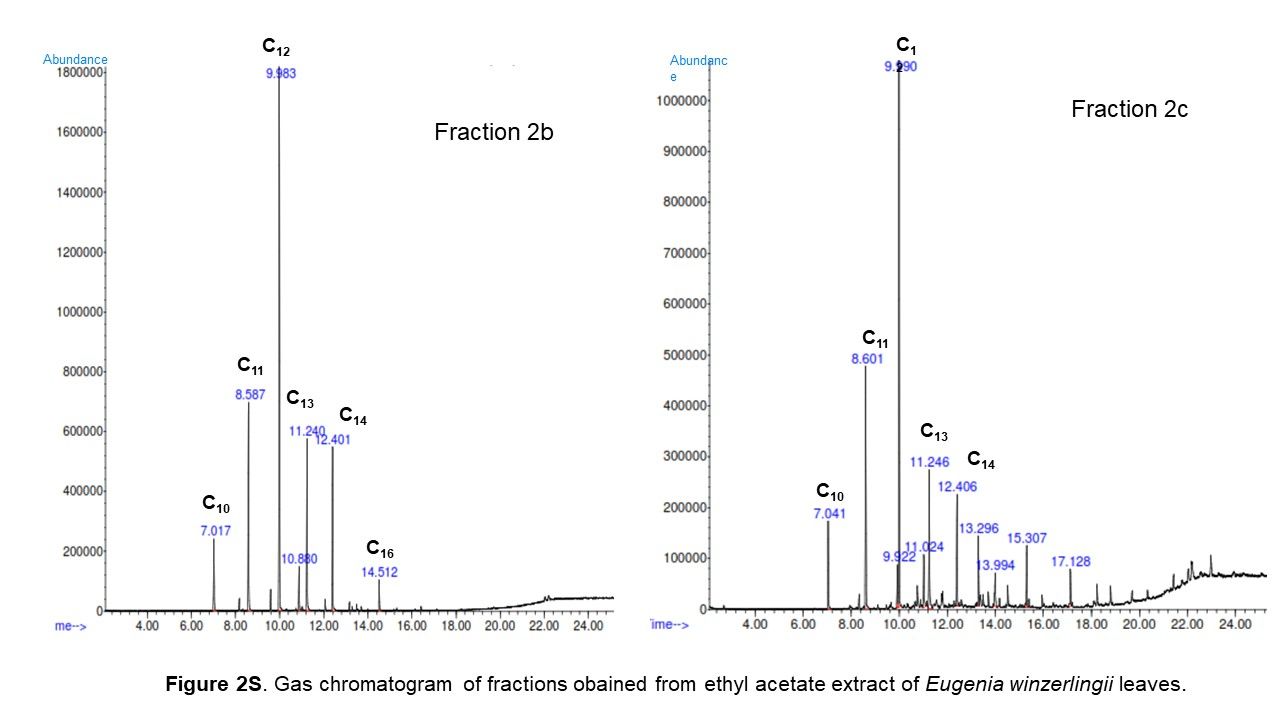

Supplement: Supplementary file 1 [file molecules-24-01724-s001.zip › Suplementary material/Figure 2S.jpg]
